# RECENT DEVELOPMENTS IN FRAILTY IDENTIFICATION, TREATMENT, AND PREVENTION IN OLDER ADULTS: A REVIEW

**DOI:** 10.1093/geroni/igad104.2796

**Published:** 2023-12-21

**Authors:** Emiel Hoogendijk, Elsa Dent

**Affiliations:** Amsterdam University Medical Centers, Amsterdam, Noord-Holland, Netherlands; Torrens University Australia, Adelaide, South Australia, Australia

## Abstract

Frailty is a clinical condition characterized by an increased susceptibility to stressors and an elevated risk of adverse outcomes such as mortality. In the light of global population aging, the prevalence of frailty is expected to soar in coming decades. The current study provides a review of novel developments and emerging practices in frailty research regarding prevention, identification, and management of the condition in older adults. We searched geriatric journals in the top two quartiles (based on 2021 Impact Factors from Clarivate Journal Citation Reports) for articles published between January 2018 and December 2022. We screened 1519 articles for eligibility and assessed the full text of 278 articles. Popular research trends included the study of frailty transitions and trajectories, analyses of life-course risk factors for frailty, identifying frailty using biomarkers (and biomarker panels), and investigating the altered response to medications by older adults with frailty. Other areas with novel developments included: artificial intelligence (machine learning) for frailty identification; exercise and physical activity for the management of frailty; medication management, gerontechnology; education of healthcare professionals; integrated care; person-centered care; guidelines; cost-effectiveness of management strategies; and COVID-19. This review identified a strong need for the implementation and evaluation of cost-effective and community-based interventions to prevent and manage frailty. Our findings highlight the need to strengthen healthcare systems to better support older adults with frailty and involve those with frailty in shared decision-making regarding their care.

